# miR-195 in human primary mesenchymal stromal/stem cells regulates proliferation, osteogenesis and paracrine effect on angiogenesis

**DOI:** 10.18632/oncotarget.6589

**Published:** 2015-12-13

**Authors:** Maria Ines Almeida, Andreia Machado Silva, Daniel Marques Vasconcelos, Catarina Rodrigues Almeida, Hugo Caires, Marta Teixeira Pinto, George Adrian Calin, Susana Gomes Santos, Mário Adolfo Barbosa

**Affiliations:** ^1^ Instituto de Investigação e Inovação em Saúde/Institute for Research and Innovation in Health (I3S), University of Porto, Porto, Portugal; ^2^ Instituto de Engenharia Biomédica (INEB), University of Porto, Porto, Portugal; ^3^ Instituto de Ciências Biomédicas Abel Salazar (ICBAS), University of Porto, Porto, Portugal; ^4^ Institute of Molecular Pathology and Immunology of University of Porto (Ipatimup), Porto, Portugal; ^5^ Department of Experimental Therapeutics, The University of Texas MD Anderson Cancer Center, Houston, TX, USA

**Keywords:** microRNAs, mesenchymal stromal/stem cells, differentiation, angiogenesis, VEGF, Gerotarget

## Abstract

Mesenchymal Stromal/Stem Cells (MSC) are currently being explored in diverse clinical applications, including regenerative therapies. Their contribution to regeneration of bone fractures is dependent on their capacity to proliferate, undergo osteogenesis and induce angiogenesis. This study aimed to uncover microRNAs capable of concomitantly regulate these mechanisms. Following microRNA array results, we identified miR-195 and miR-497 as downregulated in human primary MSC under osteogenic differentiation. Overexpression of miR-195 or miR-497 in human primary MSC leads to a decrease in osteogenic differentiation and proliferation rate. Conversely, inhibition of miR-195 increased alkaline phosphatase expression and activity and cells proliferation. Then, miR-195 was used to study MSC capacity to recruit blood vessels *in vivo*. We provide evidence that the paracrine effect of MSC on angiogenesis is diminishedwhen cells over-express miR-195. VEGF may partially mediate this effect, as its expression and secreted protein levels are reduced by miR-195, while increased by anti-miR-195, in human MSC. Luciferase reporter assays revealed a direct interaction between miR-195 and VEGF 3′-UTR in bone cancer cells. In conclusion, our results suggest that miR-195 regulates important mechanisms for bone regeneration, specifically MSC osteogenic differentiation, proliferation and control of angiogenesis; therefore, it is a potential target for clinical bone regenerative therapies.

## INTRODUCTION

During bone regeneration, three main phases, inflammation, new bone formation and bone remodeling take place in an orchestrated fashion to repair the injury. Human multipotent Mesenchymal Stromal/Stem Cells (MSC) are progenitor cells capable to differentiate into multiple cell lineages, including bone, cartilage, fat, and muscle [1, 2]. MSC are present in bone marrow and in several connective tissues [1, 2] and can be isolated and expanded *in vitro*. This, together with their paracrine effect on immune response modulation and angiogenesis stimulation, makes MSC attractive candidates for clinical applications [1]. Patients suffering from delayed or impaired healing, which accounts for 5-10% of patients with bone fractures [3, 4], as well as patients who suffer from bone diseases such as osteoporosis, could potentially benefit from MSC-associated therapies [5, 6, 7]. Consequently, modulation of gene expression of MSC at the bone fracture site is emerging as an attractive approach for the improvement of bone fracture healing and for the therapy of bone-related diseases [5, 8]. Such strategy would be particularly important for the elderly population who not only have an increased risk of bone fractures but also have their bone regenerative capacity compromised [9].

MicroRNAs (miRNAs) are small non-coding RNAs, approximately 22-nucleotides (nt) long, that control gene expression at the post-transcription level by binding to 5′untranslated region (UTR), coding regions or 3′UTR of messenger RNAs (mRNA). Upon binding, miRNAs inhibit mRNA translation or cause mRNA degradation [10]. miRNAs regulate virtually all cellular mechanisms and are deregulated in several human diseases, including cancer, neurodegenerative diseases, autoimmune diseases, among others [10]. Proof-of-concept of pre-clinical application of miRNAs was achieved by the demonstration that a locked nucleic acid-modified antisense oligonucleotide designed against miR-122 was able to suppress hepatitis C virus (HCV) viremia without side effects in primates [11]. Later, anti-miR-122 therapy was tested in human HCV patients and resulted in a dose-dependent reduction in HCV RNA levels and no adverse effects [12], opening an avenue for the use of miRNA-associated therapies in humans. In regenerative medicine, miRNA therapies are not yet translated into the clinics but are an emerging and exciting area of research [13]. Interestingly, modulation of a single miRNA can improve treatments. This effect may be explained by the fact that a single miRNAs is able to regulate a large number of mRNAs. Consequently, manipulation of the expression of a single miRNA may affect simultaneously several biological processes, including osteogenesis and angiogenesis, which are two coupled mechanisms absolutely required for bone formation [14, 15, 16, 17].

The aim of this work is to identify miRNAs that orchestrate crucial events in bone regeneration/repair. By modulating the levels of a single miRNA we intend to act synergistically on central functions for bone regenerative therapies, including MSC osteogenic differentiation, MSC proliferation and MSC ability to control angiogenesis.

## RESULTS

### microRNA expression during osteogenic differentiation of primary human MSC and mouse MC3T3 cell line

With the aim to explore miRNAs involved in the bone regeneration process, we started by performing a miRNA expression profile by microarrays along osteogenic differentiation. In order to obtain sufficient material and a representative pattern MC3T3, a pre-osteoblast cell line, was used for the array.

**Figure 1:**
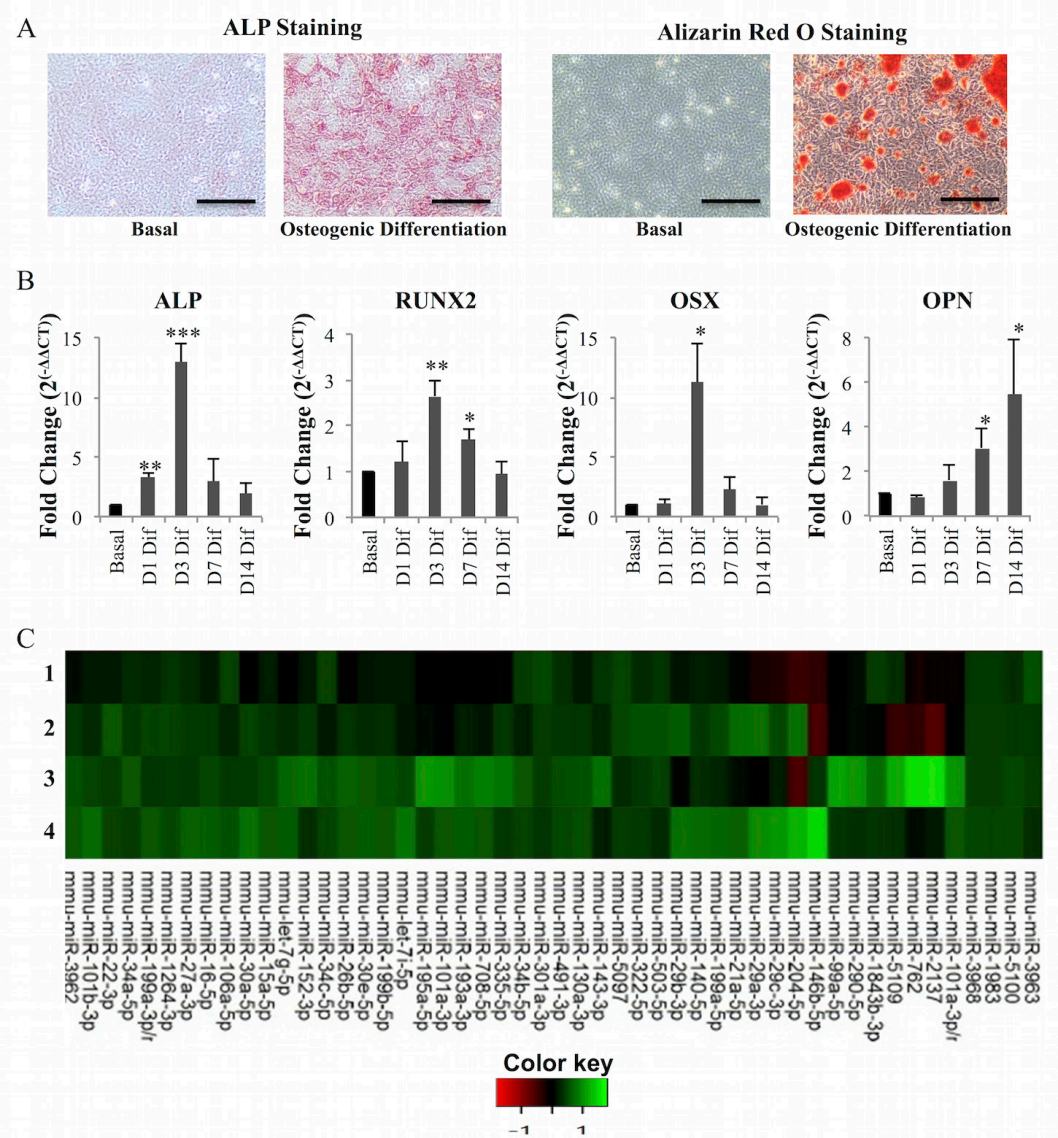
microRNA levels are altered during osteogenesis Osteoblast differentiation was induced in MC3T3 cells with dexamethasone, β-glycerophosphate and ascorbic acid. **A.** ALP staining detected ALP activity in cells grown with osteogenic differentiation but not with basal media (without osteogenic differentiation supplements); Alizarin Red S staining detected presence of calcium deposits (mineralization) in cells grown in osteogenic differentiation media but not in basal media (10X; scale: 100 μm). **B.** ALP, RUNX2, OSX and OPN mRNA levels were measured by quantitative real-time PCR. GAPDH was used as reference control. Expression levels at day 1 (D1), day 3 (D3), day 7 (D7) and day 14 (D14) of differentiation (dif) were normalized to expression levels of cells grown in basal conditions for the same time points (mean±SD, *N* = 4; *P < 0.05, **P < 0.01, ***P < 0.001, Student *t* test). **C.** Heatmap diagram of microRNA microarray data. Each column represents a microRNA and each row represents a sample: 1- day 3 basal; 2- day 3 osteogenic differentiation; 3- day 7 basal; 4- day 7 osteogenic differentiation (Basal: without osteogenic supplements, Osteogenic differentiation: with osteogenic supplements). The color scale illustrates the relative expression level of microRNAs. Red color represents an expression level below the reference channel, and green color represents expression higher than the reference.

To confirm osteogenic differentiation, cultures were set up in parallel to follow the classical osteogenic differentiation markers. Results obtained show, as expected, that after 7 days stimulating cells with osteogenic-inducing supplements, activity of Alkaline Phosphatase (ALP, a membrane-bound metalloenzyme which is considered an early osteogenic differentiation marker) was strongly increased, visible as the formation of a red insoluble product after incubation with Fast Violet B salt /Naphthol AS-MX (Figure 1A). Also, after 14 days of osteogenic differentiation, MC3T3 cells exhibited calcium-rich deposits (mineralization) visualized upon Alizarin Red S staining (Figure 1A). ALP activity and mineralization were not detected in cells grown without osteogenic-inducing supplements (basal condition) (Figure 1A). Furthermore, to confirm osteogenic gene expression program, expression levels of key genes directing bone formation were analyzed by RT-qPCR: ALP, RUNX2 and OSX expression levels were upregulated, particularly at day 3 of the differentiation process, while the late osteogenic marker OPN was upregulated after 7 and 14 days of culture with osteogenic-inducing supplements (Figure 1B). Cultures in the same conditions were used to perform a miRNA expression profile by microarrays, at days 3 and 7 of differentiation, which was compared with the miRNA profile of cells grown under basal conditions (Figure 1C). In total, 358 miRNAs were analyzed.

**Figure 2:**
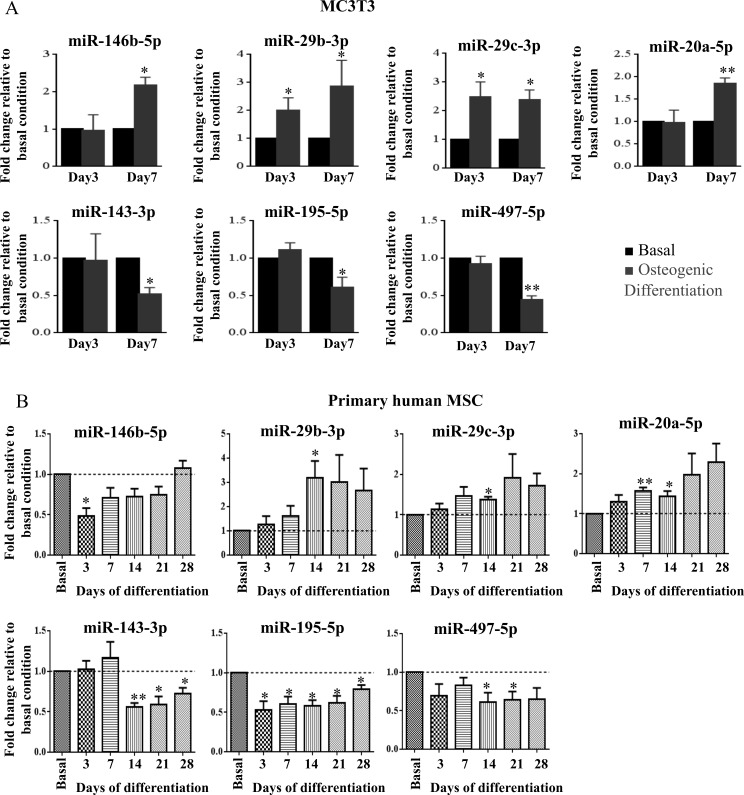
miR-146b-5p, miR-29b-3p, miR-29c-3p, miR-20a-5p, miR-143-3p, miR-195-5p and miR-497-5p expression levels were measured by quantitative real-time PCR Small nuclear RNA U6 was used as a reference gene **A.** miRNA expression levels of MC3T3 cells grown under osteogenic differentiation stimuli (osteogenic differentiation) were compared with miRNA expression levels of cells grown without osteogenic differentiation stimuli (basal), after 3 and 7 days in culture. Values are representative of 3 independent experiments (mean±SD; *P < 0.05, **P < 0.01, Student *t* test). **B.** miRNA expression levels of 4 primary human MSC grown under osteogenic differentiation stimuli (osteogenic differentiation) were compared with miRNA expression levels of cells grown without osteogenic differentiation stimuli (basal), after 3, 7, 14, 21 and 28 days in culture (mean±SEM; *P < 0.05, **P < 0.01, Student *t* test).

Considering absolute values of log fold change larger than 1, seven miRNAs (mmu-miR-2137, mmu-miR-204-5p, mmu-miR-762, mmu-miR-146b-5p, mmu-miR-711, mmu-miR-222-3p, mmu-miR-25-5p) were differently expressed after 7 days, while two miRNAs (mmu-miR-3473b, mmu-miR-204-5p) were differently expressed after 3 days of osteogenic differentiation *versus* basal conditions at the same time points (Supplementary Table 1). As small differences in miRNA levels can have a major impact on cell mechanisms and phenotypes, for array validation we not only considered high differently expressed miRNAs but we also randomly selected miRNAs with log fold change larger than 0.4. In total, ten miRNAs were selected for validation in 3 independent experiments using RT-qPCR. During MC3T3 osteogenic differentiation, miR-29b-3p, miR-29c-3p and miR-204 expression was upregulated, both at day 3 and at day 7 of differentiation, while miR-146b-5p and miR-20a-5p were upregulated only at day 7 of differentiation, which is in agreement with microarray results (Figure 2A; Supplementary Figure 1). On the other hand, miR-143-3p, miR-195a-5p and miR-497-5p expression was downregulated in osteo- *versus* basal condition at day 7 but not at day 3 (Figure 2A). These differences are consistent with the array results (Supplementary Table 1). Conversely, miR-33a-5p and miR-711 were expressed at very low levels with Cq values higher than 35 cycles and, consequently, array results were not confirmed for these miRNAs.

Although some miRNAs are conserved through evolution, their regulation and target genes may differ from mouse to human, so it was fundamental to determine miRNAs expression during human MSC osteogenic differentiation. For this purpose, bone marrow derived primary human MSC from different donors were used. Cells were incubated with osteogenic supplements and allowed to differentiate up to 28 days. Differentiation was confirmed, as in osteogenic-supplemented cells ALP activity increased, mineralization was present and ALP gene expression increased, compared with cells grown in basal conditions (Supplementary Figure 2). miRNA expression levels in osteogenic stimulated primary human MSC were compared with basal control (without osteogenic supplements) at 5 different time points (3, 7, 14, 21, and 28 days). Results obtained show that miR-29b-3p, miR-29c-3p and miR-20a-5p were upregulated upon osteogenic differentiation, while miR-143-3p, miR-195-5p and miR-497-5p were downregulated (Figure 2B). Notably, downregulation of miR-195-5p was consistent and significant along all differentiation time points (Figure 2B). Upregulation of miR-146b-5p and miR-204 was not demonstrated in primary human MSC osteogenesis (Figure 2B, Supplementary Figure 1). The levels of miR-33a-5p and miR-711 in primary human MSC were undetectable by RT-qPCR.

Among the analyzed miRNAs, miR-195-5p (and the genomic closely located miR-497-5p - Supplementary Figure 3) were selected for further analysis since 1) this is the first report showing downregulation of miR-195-5p in human primary MSCs under osteogenic differentiation conditions *versus* basal control, 2) the profile is consistent for all MSC donors during 28 days, and 3) other biological mechanisms for this miRNA in primary human MSC are still unexplored.

### miR-195 and miR-497 modulates osteogenic differentiation in primary human MSC and mouse MC3T3 cell line

**Figure 3:**
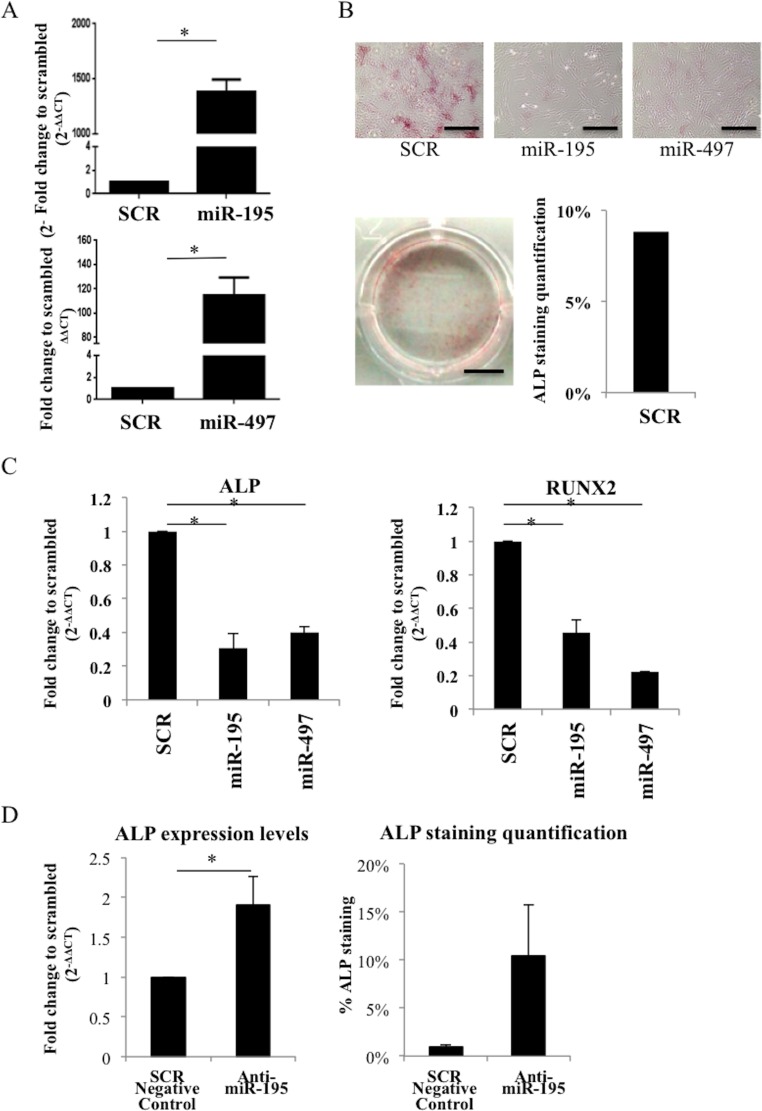
miR-195 and miR-497 decrease osteogenesis in human primary MSC **A.** miR-195 and miR-497 levels after electroporation of human MSC with miR-195 and miR-497 were determined by quantitative real time PCR. Small nuclear RNA U6 was used as reference gene. Values shown represent 2 independent experiments and are relative to scrambled negative control (SCR). **B.** ALP staining 7 days after electroporation of human MSC with scrambled negative control (SCR), miR-195 or miR-497 (5X; microscope scale: 50 μm; photography scale: 2 mm). **C.** ALP and RUNX2 expression levels in human MSC electroporated with either SCR, miR-195, miR-497. **D.** ALP expression levels 48h after MSC transfection with SCR negative control or anti-miR-195; ALP staining quantification in MSC transfected with SCR negative control or anti-miR-195 after 5 days of incubation with osteogenic supplements. Values shown represent 2 independent experiments and are relative to SCR (mean±SD; *P < 0.05, Student *t* test).

To further elucidate the impact of miR-195 and miR-497 during osteogenic differentiation of human primary MSC, these were successfully electroporated with SCR control, miR-195 or miR-497 (*P* < 0.05) (Figure 3A) and allowed to differentiate with osteogenic supplements up to 7 days. miR-195 and miR-497- overexpressing cells did not show ALP activity (negative for ALP staining) while SCR control MSC showed positive ALP staining in 9% of the well surface (Figure 3B). This result is in agreement with the significant reduction in ALP mRNA expression level in miR-195 and in miR-497-electroporated cells compared with SCR control (*P* < 0.05) (Figure 3C). Moreover, expression levels of the key osteogenic differentiation marker RUNX2 was diminished in miR-195 and miR-497-electropotared cells compared with SCR control (*P* < 0.05) (Figure 3C). To further test the specific effect of miR-195 on osteogenesis, we successfully transfected cells with anti-miR-195 (Supplementary Figure 4) and analyzed its effect on the early osteogenic marker ALP. Results showed a significant increase of 1.9-fold in ALP expression level (*P* < 0.05) and enhanced ALP activity in anti-miR-195-MSC when compared with control, 5 days after stimulation with osteogenic differentiation supplements (Figure 3D). This result further strengthens the impact of miR-195 on osteogenic differentiation. Therefore, we concluded that miR-195 and miR-497 play an anti-osteogenic differentiation role in human primary MSC cells.

The same effect was observed during osteogenic differentiation of MC3T3. Cells were transfected with either SCR, miR-143 (used as a positive control), miR-195 or miR-497. ALP staining levels decreased to 21% and 60% in miR-195 and miR-497 transfected cells, respectively, compared with SCR control (Supplementary Figure 5A). In agreement, early osteogenic differentiation markers ALP, RUNX2, OSX expression levels were decreased (Supplementary Figure 5B).

### miR-195 and miR-497 affects cell proliferation of primary human MSC

**Figure 4:**
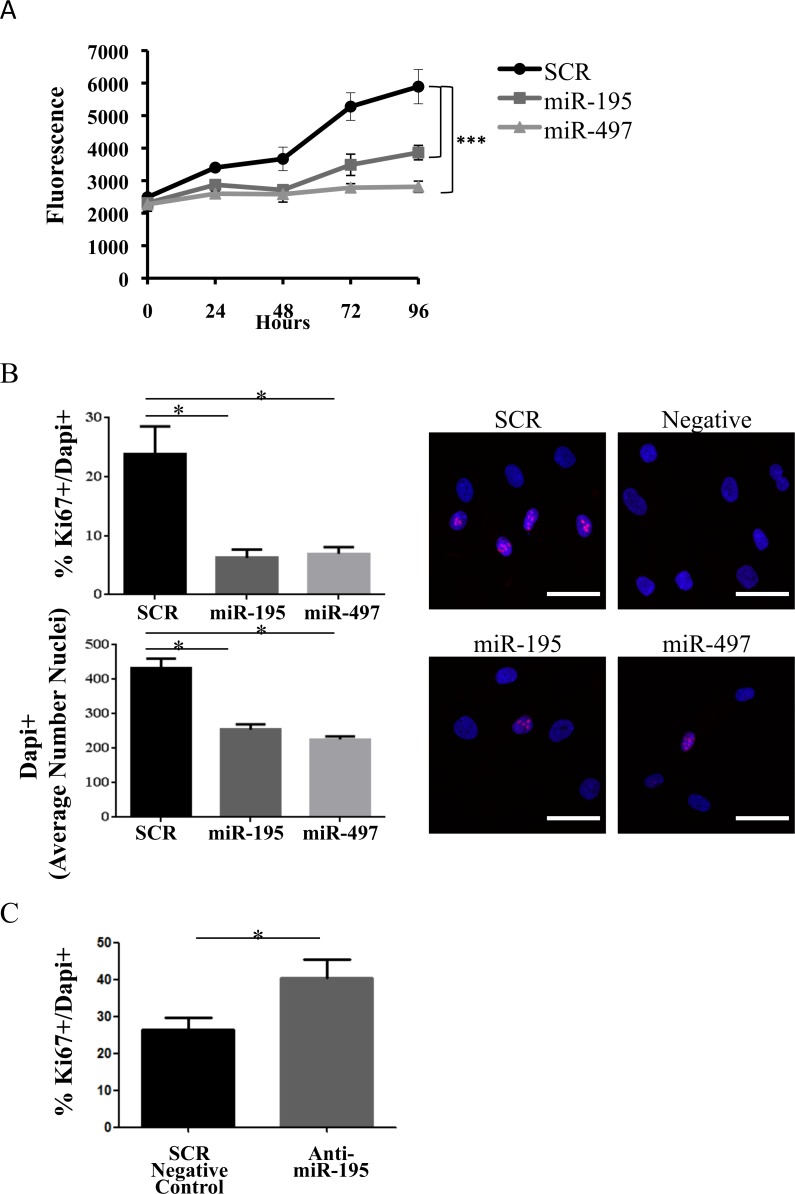
miR-195 expression impairs MSC proliferation **A.** A representative experiment on cell viability of miR-195 and miR-497 in human MSC. Fluorescence of resorufin was measured every 24 hours for 4 days post-electroporation with SCR, miR-195 or miR-497. Values represent the mean±SD of 5 replicates (***P < 0.001, Student *t* test). Two independent experiments were performed. **B.** Confocal imaging of MSC cells 48 hours after electroporation of SCR, miR-195 or miR-497. Cells were stained for the proliferation marker Ki-67 protein (red); nuclear DNA was labeled with DAPI (blue). Differences in the percentage of cells in proliferation (Ki-67+/DAPI+) and the average number of nuclei (DAPI+) were determined in 2 independent experiments by analyzing in at least 5 different images per condition with a minimum of 100 DAPI+ nuclei each (*P < 0.05, one-way ANOVA). Laser Scanning Spectral Confocal Microscope Leica TCS SP2 was used. A representative merged fluorescence image (20X objective, zoom factor of 5) per condition is shown (DAPI+ - blue; Ki-67+ - red). Scale: 40 μm. **C.** Differences in the percentage of proliferative MSC (Ki-67+/DAPI+) 96h after transfection of SCR negative control or anti-miR-195 (*P < 0.05, one-way ANOVA).

To characterize the impact of miR-195 and miR-497 on MSC biology, we next investigated their impact on cell proliferation. The resazurin reduction assay, where active viable cells reduce the non-fluorescent dye resazurin to the strongly-fluorescent dye resorufin, was used as an indirect assessment of cell proliferation. In two independent experiments, we measured cell viability in miR-195 and miR-497-electroporated human MSC. Cell viability was significantly decreased in miR-195 and miR-497-overexpressing MSC (*P* < 0.001) in comparison with the SCR control (Figure 4A).

To further confirm if cell proliferation was indeed affected by miR-195 and miR-497, staining with the proliferation marker Ki-67 was quantified. Ki-67 positive cells were counted and normalized to the total number of cells (DAPI positive cells). Ki-67+/DAPI+ levels were significantly reduced in miR-195 and miR-497-electroporated cells compared with SCR control 48h post-electroporation (*P* < 0.05) (Figure 4B). Therefore, miR-195 and miR-497 impaired MSC proliferation. Additionally, significantly more nuclei (DAPI+) were detected in SCR control cells (*P* < 0.05) (Figure 4B) when compared with the number of nuclei in miRNA-electroporated cells, which further supports the effect of these miRNAs on MSC proliferation. The difference in proliferative cells (Ki-67+/DAPI+) is evident even 96h after electroporation (*P* < 0.05) (Supplementary Figure 6). Furthermore, the specific effect of miR-195 on proliferation was confirmed, as treatment with miR-195 antagonist (anti-miR-195) significantly increased Ki-67+/DAPI+ levels, compared with control (*P* < 0.05) (Figure 4C).

In conclusion, our data shows that both miR-195 and miR-497 decrease cell proliferation in human primary MSC.

### Angiogenesis is decreased by miR-195 and miR-497 expression in primary human MSC

Considering that an adequate angiogenic response is crucial for successful bone healing [18] and knowing that MSC are able to recruit and induce formation of new vessels during bone regeneration [14, 15], we investigated the impact of miR-195 and miR-497 on this biological process.

**Figure 5:**
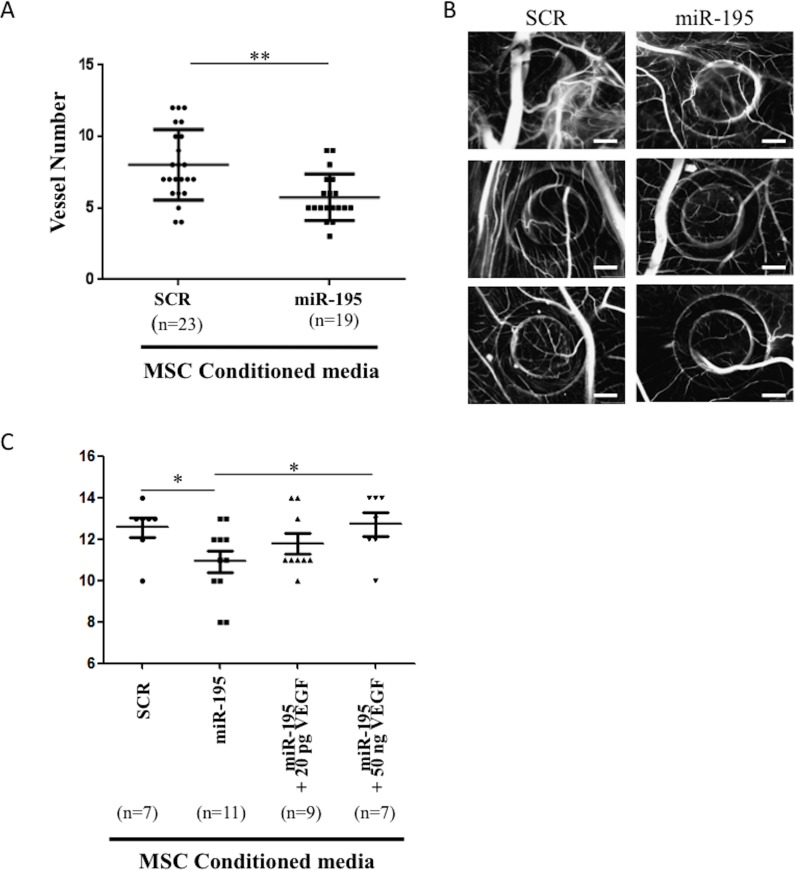
*In vivo* chick chorioallantoic membrane (CAM) assay **A.** Number of vessels in the CAM after 72 hours incubation with SCR-electroporated MSC condition media or miR-195-electroporated MSC condition media (mean±SD, **P < 0.01, Student *t* test). Graphic includes results from 3 independent replicates, in a total of 42 analyzed eggs. **B.** Three representative images are shown per condition (20X; scale: 1mm). **C.** Number of vessels in the CAM after 72 hours incubation with SCR-electroporated MSC conditioned media or miR-195-electroporataed MSC conditioned media with or without human recombinant VEGF supplementation (mean±SD, *P < 0.05, one way ANOVA), in a total of 34 analyzed eggs.

Human primary MSCs were electroporated with either miR-195 or SCR control. After 72 hours, conditioned media was collected, concentrated and placed on top of E10 growing CAM. Two independent experiments were performed and a total of 24 eggs were used per condition. Out of those, 23 eggs survived with SCR-conditioned media and 21 eggs survived with miR-195-conditioned media although only 19 allowed the reading for evaluation of the angiogenic response. Our results showed a statistically significant decrease of 28% (Mean ± SEM SCR: 8,000 ± 0,5146 *N* = 23, miR-195: 5,737 ± 0,3734 *N* = 19; *P* < 0.01) in the endothelial vessels growth induced by miR-195 MSC conditioned media compared with SCR control MSC conditioned media (Figure 5A, 5B). In conclusion, expression of miR-195 in MSC regulates the angiogenic activity of endothelial cells.

This assay was also performed using conditioned media from MSCs electroporated with either miR-497 or SCR control. Our results showed a statistically significant decrease of 12% (Mean ± SEM SCR: 10,60 ± 0,3352 *N* = 15, miR-497: 9,313 ± 0,3950 *N* = 16; *P* < 0.05) in the endothelial vessels growth induced by miR-497 MSC conditioned media compared with SCR control MSC conditioned media (Supplementary Figure 7). Taken together, the *in vivo* CAM assay results suggest miR-195 negatively impacts angiogenesis to a greater extent than miR-497.

Additionally, we tested whether VEGF, a known key angiogenic mediator [14, 15], was able to rescue the effect of this miRNA on angiogenesis *in vivo*. An independent CAM assay experiment was performed and results are shown in Figure 5C. Interestingly, VEGF was able to dose-dependently revert the decrease in the number of new blood vessels caused by miR-195-MSC conditioned media. When compared to miR-195-MSC conditioned media, low doses of VEGF produced a slight recovery, while high levels of VEGF significantly increased the number of vessels (*P* < 0.05), restoring the number of vessels formed in response to SCR control condition media (Mean ± SEM SCR: 12,57 ± 0,4809 *N* = 7, miR-195: 10,91 ± 0,5301 *N* = 11, Mean ± SEM miR-195+20pgVEGF: 11,78 ± 0,4938 *N* = 9, miR-195+50ngVEGF: 12,71 ± 0,5654 *N* = 7; SCR *vs* miR-195 and miR-195 *vs* miR-195+50ngVEGF *P* < 0.05) (Figure 5C).

### miR-195 decreased VEGF expression and MSC-secreted VEGF levels

**Figure 6:**
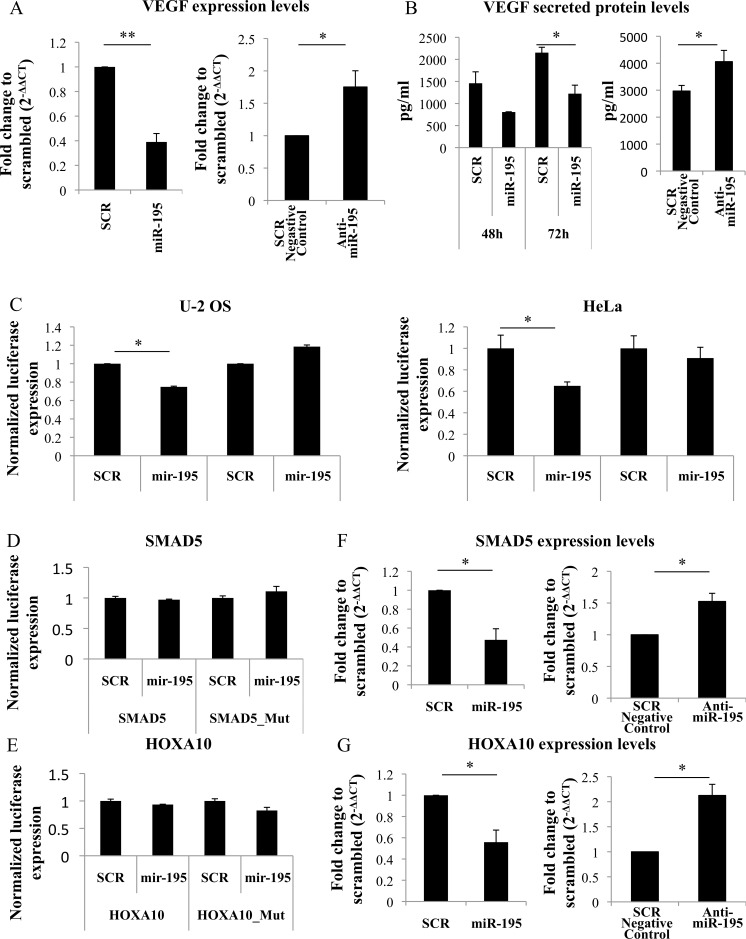
VEGF regulation by miR-195 **A.** VEGF expression levels are decreased in miR-195-overexpressing MSC compared with scrambled when analyzed by quantitative real-time PCR (**P < 0.01, Student *t* test). VEGF expression levels are increased in anti-miR-195 MSC compared with scrambled negative control when analyzed by quantitative real-time PCR (*P < 0.05, Student *t* test). **B.** Levels of secreted VEGF protein (pg/ml) were measured by enzyme-linked immunosorbent assay and are reduced in miR-195-MSC conditioned media *versus* SCR-MSC conditioned media (*P < 0.05, Student *t* test), while increased in anti-miR-195-MSC conditioned media *versus* SCR negative control-MSC conditioned media (*P < 0.05, Student *t* test). **C.** Luciferase levels were reduced when PGL3-VEGF (VEGF) but not PGL3-VEGF-mutated (VEGF_Mut) was cotransfected with miR-195 compared with scrambled (SCR), in both U-2 OS and HeLa cells. Values represent mean±SD of 3 replicates (*P < 0.05, Student *t* test). **D.** Luciferase levels were not altered after cotranfection of PGL3-SMAD5 (SMAD5) with miR-195 compared with scrambled (SCR) in U-2OS cells. **E.** Luciferase levels were not altered after cotranfection of PGL3-HOXA10 (HOX10A) with miR-195 compared with scrambled (SCR) in U-2OS cells. **F.** Expression levels of SMAD5 were decreased 48hours after MSC miR-195-electroporation, while increased 48hours after MSC anti-miR-195-transfection compared with control. Values represent mean±SD of 2 replicates (*P < 0.05, Student *t* test). **G.** Expression levels of HOXA10 were decreased 48hours after MSC miR-195-electroporation, while increased 48hours after MSC anti-miR-195-transfection compared with control. Values represent mean±SD of 2 replicates (*P < 0.05, Student *t* test).

We next investigated VEGF as a potential target for miR-195. In fact, *in silico* predictions using different algorithms indicate miR-195 targets VEGF 3′UTR (Supplementary Figure 8). To test if VEGF levels were altered by miR-195, RNA was isolated from MSC 48h after miR-195 electroporation. VEGF expression levels were significantly decreased in miR-195 MSC *versus* SCR control (*P* < 0.01) (Figure 6A). Conversely, MSC transfected with anti-miR-195 expressed higher levels of VEGF than control (*P* < 0,05) (Figure 6A).

Also, levels of VEGF secreted protein, which are important for vessels formation, were measured by ELISA. Concentration of secreted VEGF was significantly decreased in miR-195 electroporated MSC *versus* control (*P* < 0.05) (Figure 6B). On the other hand, VEGF was found to be increased in cell culture supernatants of MSC after miR-195 knockdown (*P* < 0,05) (Figure 6B).

### miR-195 directly targets VEGF

To test if miR-195 directly binds VEGF in bone cells luciferase assays were performed in U-2 OS cells. Luciferase levels were significantly reduced when cells were co-transfected with pGL3-VEGF binding site construct and miR-195 compared with control. However, this difference was abolished when the binding site was mutated suggesting a specific VEGF::miR-195 direct interaction (Figure 6C). The same results were obtained when using HeLa cells, which is a cell line commonly used in human *in vitro* studies (Figure 6C).

Also, SMAD family member 5 (SMAD5) and Homeobox A10 (HOXA10) genes, located in chromosome 5 and chromosome 7, respectively, have been described as regulators of osteogenesis and/or angiogenesis [19, 20, 21]. Both genes were predicted to be targeted by miR-195 (Supplementary Figure 8). Potential interaction site was cloned into PGL3 vector. Luciferase levels of pGL3-SMAD5 and PGL3-HOXA10 or mutated constructs co-transfected with miR-195 were not altered compared with control (Figure 6D, 6E). On the other hand, mRNA levels of SMAD5 and HOX10A were decreased after miR-195 MSC electroporation, while increased after anti-miR-195 MSC tranfection (Figure 6F, 6G), indicating that miR-195 controls SMAD5 and HOXA10 expression but it does not directly interact with SMAD5 and HOXA10. However, we cannot exclude the hypothesis of a direct SMAD5::miR-195 and HOXA10::miR-195 interaction in other mRNA sites or cells lines.

## DISCUSSION

Increased risk of bone fracture is associated with aging and the regenerative capacity is reduced in the elderly [9]. New clinical strategies are currently being developed to improve bone regeneration and, among those, modulation of miRNA levels has been investigated [13]. Beyond their role as regulators of the immune response, MSC therapeutic effect for bone regeneration is likely related to their capacity to proliferate, undergo osteogenic differentiation and control angiogenesis [22]. In this study, we analyzed the ability of miRNAs to regulate these processes.

Firstly, miRNA profiling of cells under osteogenic differentiation stimuli *versus* non-stimulated cells was obtained and then the microarray data was validated by RT-qPCR in mouse pre-osteoblasts and in human bone marrow-derived primary MSC. Upregulation of miR-20a during human osteogenic differentiation was previously described by Zhang *et al.* [23]. Therefore, we analyzed miR-20a expression in our MSC samples as a positive control. Moreover, miR-146b expression levels during MC3T3 osteogenic differentiation were upregulated compared with controls, but this profile was not confirmed for human MSC differentiation. This is not surprising as regulation of human and mouse miRNA may differ [24]. Also, miRNA targets in both species may be totally different, which reinforces the importance of studies using human primary cells. Furthermore, validation of our miRNA-microarray results by RT-qPCR confirmed miR-29b and miR-29c as positive regulators of osteoblast differentiation in the mouse cell line, which is in agreement with previous reports and further strengthen the quality/relevance the array data [25, 26]. However, studies on human MSC were missing. This is the first report demonstrating miR-29b and miR-29c upregulation during osteogenic differentiation in human primary MSC. Moreover, we demonstrated that miR-143 levels are consistently decreased in MSC after 14 days of differentiation in human cells, suggesting that this miRNA negatively regulates late mineralization stages.

Within the analyzed miRNAs, miR-195 was the only one consistently and significantly downregulated in human MSC under osteogenic stimuli compared with non-stimulated cells in all time points and, for this reason, was further explored. Interestingly, miR-195 is located within less than 10kb distance from miR-497, in chromosome 17 (sequence details are shown in Supplementary Table 2). *In vitro* results indicate both miRNA are able to suppress osteogenic differentiation in human primary MSC. A recent study by Grünhagen *et al.* showed that miR-195-miR-497 cluster impaired osteoblast differentiation in mouse cells [27]. Thus, in general terms, the miR-195 and miR-497 regulation of osteogenic differentiation are likely conserved from mouse to human.

However, different biological processes beyond MSC differentiation contribute to bone regeneration, and differences in a single miRNA may affect simultaneously distinct biological mechanisms [28]. So, the potential involvement of miRNA in MSC proliferation and their capacity to promote angiogenesis were also studied. Gain-of-function studies revealed miR-195 (and the clustered miR-497) decreased cell proliferation, as determined by significant decreases in the number of positive cells for the cellular proliferation marker Ki-67, which is present during all active phases of the cell cycle, and in the overall number of cells. As expected, miR-195 knockdown increased MSC proliferation. Growth-suppressive properties of miR-195 have been mainly described for tumor cells, particularly for hepatocellular carcinoma [29, 30], esophageal squamous cell carcinoma [31], non-small cell lung cancer [32, 33, 34] and colorectal cancer [35]. To the best of our knowledge our study is the first to describe the role of miR-195 in the proliferation of human primary non-malignant and non-transformed cells. This effect in not likely to be explained by extracellular VEGF as no differences in MSC proliferation were found when recombinant VEGF was added to our MSC primary cultures (data not shown). Considering that this miRNA has multiple targets, some with potential effects on cell proliferation, it is reasonable to expect that this effect may be mediated by other miR-195 targets acting intracellularly (e.g. SMAD5 or HOXA10 related pathways).

Formation of new blood vessels is an essential process for tissue regeneration, as it mediates the transport of nutrients, oxygen, growth factors and circulating cells [36, 37]. For this reason, the effect of miR-195 on angiogenesis was tested *in vivo*, using the chicken CAM assay model. We concluded that miR-195 decreased significantly the number of vessels formed in response to MSC conditioned medium. This biological effect is crucial for bone formation and may be partially explained by the ability of miR-195 to target VEGF. Moreover, other proteins in the supernatants, which could be miR-195 direct or indirect targets, may have a synergistic effect on angiogenesis inhibition.

Interestingly, coupling of angiogenesis and osteogenesis is supported by several reports [16, 17]. *In vitro*, inhibition of VEGF decreases nodule formation and ALP activity in primary human osteoblasts [38]. *In vivo*, VEGF stimulates repair of femoral fracture and cortical bone defect in mice model and radius critical size defect in rabbit model [38]. Additionally, MSC with reduced expression of VEGF inhibited osteoblast differentiation by decreasing RUNX2 levels and mice with low VEGF expression in osteoblastic lineage cells lead to reduced bone density in mice [39]. In agreement, VEGF expression is reduced during aging [40].

In conclusion, the present study demonstrates that miR-195 (and the clustered miR-497 gene) acts as a negative regulator of osteogenesis in human primary bone marrow MSC, an inhibitor of MSC proliferative capacity and an anti-angiogenic player. All together, these results indicate miR-195 as a potential target for enhancing regeneration of human bone defects (or bone-degenerative diseases, such as osteoporosis). Efficient *in vivo* therapeutic delivery and specific cellular uptake of miRNAs antagonists for clinical application is in progress [41]. Considering the biological effect and targets of miR-195, future studies should address *in vivo* delivery of anti-miR-195 oligonucleotides into bone injury scenarios.

## MATERIALS AND METHODS

### Human mesenchymal stem/stromal cells

Primary human MSC were isolated from bone marrow collected at Hospital de S. João (Porto, Portugal) from discarded bone tissues of patients undergoing total hip arthroplasty, less than 60 years old and who did not suffer from known inflammatory diseases, according to Almeida CR et al. [42] (Supplementary Methods). Procedures were approved by the ethics committee and informed consent was obtained from all donors included in this study. Isolation and purity of MSCs was confirmed by flow cytometry after staining of surface CD105, CD73, CD90 (positive markers) and CD45, CD34, CD14, CD19 and HLA-DR (negative markers), and by testing cells ability to differentiate in osteoblasts, chondroblasts or adipocytes [17, 43].

Commercial human primary MSC for miRNA electroporation were acquired from Lonza.

### Cell lines

Pre-osteoblast mouse cell line MC3T3-E1 subclone 4 (ATCC) was grown in alpha- Minimum Essential Medium (Gibco). U-2 OS and HeLa cells (ATCC) were grown in McCoy's 5a Modified Medium (Corning Cellgro) and DMEM, respectively. Cells were incubated at 37°C and 5% CO_2_.

### Osteogenic differentiation

Cells were incubated in the presence of the osteogenic supplements 10^−7^M dexamethasone (Sigma-Aldrich), 10^−2^M β-glycerophosphate (Sigma-Aldrich) and 5×10^−5^M ascorbic acid (Sigma-Aldrich) (differentiation media) during 28 days (for human MSC differentiation into osteoblasts) or 14 days (for MC3T3 differentiation into osteoblasts). Media was changed every 3 days. ALP staining (a key osteogenic marker) was performed at day 14 (human MSC) and day 7 (MC3T3) of differentiation; Alizarin Red S (Sigma-Aldrich) staining to confirm the presence of extracellular calcium deposits (a hallmark of mineralization) was performed at day 28 (human MSC) and day 14 (MC3T3) of differentiation (Supplementary Methods).

Weak and strong ALP intensities were counted after thresholding colour images on the total area of each well and quantified as previously described by us (light ALP: red > 77 and 1.2 x green < red < 1.5 x green; strong ALP: red > 77 and red > 1.5 x green) [44].

### RNA isolation

RNA from human primary cells and cell lines was isolated using TRIzol reagent (Invitrogen), according to the manufacturer's instructions. RNA quantity was assessed with NanoDrop ND-1000 (Thermo Fisher Scientific, Wilmington, DE). RNA integrity was assessed by gel electrophoresis of total RNA ran in 1% agarose gel. RNA quality for microarrays experiment was evaluated by Experion Automated Electrophoresis System.

### Reverse transcription and real-time quantitative polymerase chain reaction (RT-qPCR)

RNA was treated with TURBO DNA-free Kit (Life Technologies) and complementary DNA (cDNA) was synthesized using Random Hexamers (Invitrogen, Life Technologies), dNTPs (Bioline) and SuperScript^®^ III Reverse Transcriptase (Invitrogen, Life Technologies). qPCR was carried out in iQ5 Real-Time PCR Detection System (Bio-Rad) using cDNA, primers and iQ SYBR Green Supermix (Bio-Rad). Primers for qPCR experiments are shown in Supplementary Table 3.

miRNA expression was evaluated using TaqMan miRNA assays (Life Technologies). Briefly, cDNA was synthesized using 20 ng of RNA as a template, gene-specific stem-loop Reverse Transcription primer, and the TaqMan microRNA reverse transcription kit (Life Technologies). qPCR was carried out in iQ5 Real-Time PCR Detection System (Bio-Rad) using cDNA, TaqMan probe and SsoAdvanced™ Universal Probes Supermix (Bio-Rad). Small nuclear RNA U6 was used as reference gene.

Experiments were performed in duplicate. Relative expression levels were calculated using the quantification cycle (C_q_) method, according to MIQE guidelines [45].

### Microarrays

MiRNA expression profiling data was generated by MicroRNA Array Services from Exiqon. Briefly, the samples were labeled using the miRCURY LNA™ microRNA Hi-Power Labeling Kit, Hy3™/Hy5™ and hybridized on the miRCURY LNA™ microRNA Array (7th Gen). Following normalization of the quantified signals (corrected background) using the global Lowess regression algorithm, an unsupervised as well as supervised data analysis was performed.

### Electroporations and transfections

Human primary MSC (Lonza) (0.5×10^6^) were mixed with Pre-miR miRNA Precursors miR-195, Pre-miR miRNA Precursors miR-497 or Pre-miR miRNA Precursor Negative Control (Scrambled - SCR) (Life Technologies) in an electroporation cuvette and electroporated using OPTI-MEM I (Invitrogen, Life Technologies) in a Gene Pulser Xcell Electroporation Systems (Bio-Rad) with the following conditions: voltage - 250 V, capacitance - 950 μF, resistance - 200 Ω [46].

For miR-195 antagonist experiments, 100nM anti-miR-195 (mirVana^®^ miRNA inhibitor, ThermoFisher Scientific) or SCR Negative Control (Anti-miR Negative Control #1, ThermoFisher Scientific) were used to transfect MSC using Lipofectamine 2000 reagent (Invitrogen), according to manufacturer's instructions.

MC3T3 cells (3×10^6^) plated in a 10-cm culture dish were transfected with 50 nM Pre-miR miRNA Precursors miR-143, Pre-miR miRNA Precursors miR-195, Pre-miR miRNA Precursors miR-497 or Pre-miR miRNA Precursor Negative Control (Scrambled - SCR) (Life Technologies) using Lipofectamine 2000 transfection reagent (Invitrogen, Life Technologies). Media without antibiotics was used.

Cells were collected after 12 hours and plated for the different assays. All Electroporations/Transfections were confirmed by RT-qPCR.

### Resazurin reduction assay

To determine the effect of miRNAs on proliferation, MSC (Passage 7) electroporated with Pre-miR miRNA Precursors miR-195, Pre-miR miRNA Precursors miR-497 or Pre-miR miRNA Precursor Negative Control (Scrambled - SCR) were plated in 96-well plates in sextuplicates. Cells were incubated with resazurin (Sigma Aldrich) for 2 h at 37°C protected from light to allow cells to convert resazurin to resorufin. Fluorescence was measured (530 nm excitation and 590 nm emission) in a Synergy HT Multi-Mode Microplate Reader (BioTek). Two independent experiments were performed. Student *t* test was used to calculate significance. Values of *P* < 0.05 were considered to be statistically significant.

### Immunostaining

miR-195, miR-497 or SCR-electroporated MSC, and anti-miR-195 or SCR negative control-transfected MSC, were grown on top of cover-slips. Cells were fixed with 4% PFA, permeabilized with 0.1% triton X-100 and blocked with 5% BSA. Cells were incubated for 2 h with primary anti-Ki-67 (Thermo Scientific) antibody and for 45 min with secondary anti-rabbit CY3 (Jackson ImmunoResearch) antibody. Vectshield with DAPI (Vector Laboratories) was used as mounting media. Slides were observed using Leica TCS SP2 (Inverted microscope Leica DMIRE2 and LCS 2.61 software). Details on quantification of total number of cells and Ki-67+ cells are provided in Supplementary Methods.

GraphPad Prism statistical program was used for the construction of a column bar graphic. One-way ANOVA (and non-parametric) was used to calculate significance. Values of *P* < 0.05 were considered to be statistically significant.

### Chicken embryo chorioallantoic membrane (CAM) angiogenesis assay

The chicken embryo chorioallantoic membrane (CAM) model was used to evaluate angiogenic effect of miR-195, miR-497 and SCR electroporated MSC conditioned media. The media was collected 72h after cell electroporation and 2x concentrated in Savant SpeedVac Concentrator under vacuum (Thermo Scientific). Fertilized chick (Gallus gallus) eggs obtained from commercial sources were incubated horizontally at 37.8°C in a humidified atmosphere and referred to embryonic day (E). On E3 a square window was opened in the shell after removal of 2-2.5ml of albumen to allow detachment of the developing CAM. The window was sealed with a transparent adhesive tape and the eggs returned to the incubator. Conditioned medium from miR-195, miR-497 or SCR expressing MSC, were placed on top of E10 growing CAM into a 3mm silicone ring under sterile conditions. The eggs were re-sealed and returned to the incubator for an additional 72h. After removing the ring, the CAM was excised from the embryos and photographed *ex ovo* under a stereoscope at 20x magnification (Olympus, SZX16 coupled with a DP71 camera). The number of new vessels (less than 15μm diameter) growing radially towards the ring area was counted in a blind fashion manner by two independent observers. The first CAM assay experiment was performed using 3 different batches of eggs and using condition media from 2 independent experiments. The second CAM assay independent experiment was performed using a new batch of eggs and 20 pg or 50 ng of human recombinant VEGF (VEGF-165, PeproTech) were added to the miR-195-MSC condition media and placed on CAM together with the controls.

GraphPad statistical program was used for data analysis. A student *t* test (for samples with unequal variance) was used to calculate significance. Values of *P* < 0.05 were considered to be statistically significant.

### Enzyme-linked immunosorbent assay (ELISA)

VEGF protein levels in supernatants of miR-195 or SCR electroporated MSC and of anti-miR-195 or SCR negative control transfected MSC were quantified using Raybiotech Human VEGF ELISA kit (Tebu-Bio), following the manufacturer protocol (Supplementary Methods). Absorbance was read at 450 nm in Synergy HT Multi-Mode Microplate Reader (BioTek). Two independent experiments were performed. Student *t* test was used to calculate significance. Values of *P* < 0.05 were considered to be statistically significant.

### *In silico* target prediction

*In silico* predictions of miR-195 targets were performed by screening the databases TargetScan (http://www.targetscan.org/), RNA22 (http://cbcsrv.watson.ibm.com/) and DIANA-microT-CDS - v5.09 (http://diana.imis.athena-innovation.gr/). miR-195 and miR-497 sequence annotations were obtained from the miRBase database (http://www.mirbase.org/) (Supplementary Table 2).

### Cloning and luciferase assays

Cloning and luciferase assays were performed as previously described by us [28] (Supplementary Methods). Primers for fragment amplification containing XbaI restriction enzyme site are described in Supplementary Table 1. Mutations on the miRNA-binding site were generated using QuikChange II XL site-directed mutagenesis kit (Agilent Technologies) (Supplementary Table 1). Luciferase activity was measured using a dual-luciferase reporter assay system (Promega Corporation), according to the manufacturer protocol, in the veritas microplate luminometer (Turner Biosystems).

## SUPPLEMENTARY MATERIAL FIGURES AND TABLES


