# Supplementary material for: miR-195 in human primary mesenchymal stromal/stem cells regulates proliferation, osteogenesis and paracrine effect on angiogenesis
Source: Oncotarget. 2015 Dec 13;7(1):7–22. doi: 10.18632/oncotarget.6589 (PMC4807979; doi:10.18632/oncotarget.6589)
Supplement: Supplementary file 1 [file oncotarget-07-0007-s001.pdf]

miR-195 in human primary mesenchymal stromal/stem cells regulates proliferation, osteogenesis and paracrine effect on angiogenesis

Supplementary Material

Supplementary Methods

Isolation and expansion of Human Mesenchymal Stem/Stromal Cells

After bone marrow collection, nucleated cells were isolated after lymphoprep gradient density centrifugation and plated in Dulbecco's modified Eagle's medium (DMEM) with low glucose and with Glutamax (Life Technologies) plus 10% selected heat inactivated Fetal Bovine Serum (Life Technologies) and 1% penicillin/streptomycin (Life Technologies). Cells were incubated at 37°C and 5% CO_2. After 72 hour (h) non-adherent cells were removed and new medium was added. Cells were allowed to grow until 80% confluence, detached with 0.05% trypsin/EDTA (Life Technologies) and grown in 150 cm^2 tissue culture flasks (BD Falcon) [42]. Cells in passages 4 to 7 were used for the experiments. Serum used for MSC differentiation was previously tested (Biowest).

ALP and Alizarin staining

Cells were fixed with 4% paraformaldehyde (PFA) (Sigma-Aldrich) for 20 min at room temperature and washed. For ALP staining, Naphthol AS-MX Phosphate alkaline solution (Sigma-Aldrich) was added to 0,025% Fast Violet B salt solution (Sigma-Aldrich), the solution was filtered and incubated with the cells for 45 minutes (min) at room temperature protected from light. For Alizarin staining, freshly made 1% alizarin red solution was incubated with the cells for 10 min at room temperature protected from light. Flasks/wells were washed with water,

photographed and visualized under a light microscope. Simultaneously, cells grown in basal conditions (without osteogenic supplements) were used as a control.

Immunostaining quantification

The number of Ki-67$^+$ cells was quantified using an image analysis algorithm developed in house using Matlab R2012b. Images acquired with 10x objective and composed by two channels containing DAPI and Ki-67 fluorescence were pre-processed using a Gaussian filter to reduce noise. Cell nuclei were detected in DAPI channel after image binarization by Otsu's method and imposing size limits from 10 pixels to 200 pixels. The total number of cells was determined counting the number of isolated objects. Ki-67 channel was also binarized using threshold level of 0,25, empirically defined by two independent and qualified users, to remove low fluorescence intensity background. Finally, DAPI and Ki-67 binarized images were intercepted to select and count (as previously performed in DAPI channel) the cell nuclei with a positive signal in Ki-67 channel.

Cloning and Luciferase assays

A fragment about 200 nucleotides (nt) comprising the predicted region of miRNA-mRNA target interaction was cloned downstream of the firefly luciferase reporter gene of the pGL3 control vector (Promega Corporation) into the XbaI site.

Cells were seeded on 24-plates and co-transfected with miR-195 or SCR (Life Technologies) and pGL3-putative binding site plasmids or pGL3-mutated putative binding site plasmids, together with Renilla luciferase construct that was used as a normalization reference. Transfections were performed in OPTI-MEM I (Invitrogen) using Lipofectamine 3000

transfection reagent (Life Technologies) for U-2 OS cells or Lipofectamine 2000 transfection reagent (Life Technologies) for HeLa cells. After 48h, transfection was stopped by adding Passive Lysis Buffer (Promega Corporation).

Osteogenic Differentiation

Cells were incubated in the presence of the osteogenic supplements 10^{-7}M dexamethasone (Sigma-Aldrich), 10^{-2}M β-glycerophosphate (Sigma-Aldrich) and 5×10^{-5}M ascorbic acid (Sigma-Aldrich) (differentiation media) during 28 days (for human MSC differentiation into osteoblasts) or 14 days (for MC3T3 differentiation into osteoblasts). Media was changed every 3 days. Alkaline Phosphatase (ALP) staining (a key osteogenic marker) was performed at day 14 (human MSC) and day 7 (MC3T3) of differentiation; Alizarin Red S (Sigma-Aldrich) staining to confirm the presence of extracellular calcium deposits (a hallmark of mineralization) was performed at day 28 (human MSC) and day 14 (MC3T3) of differentiation (Supplementary Methods).

Weak and strong ALP intensities were counted after thresholding colour images on the total area of each well and quantified as previously described by us (light ALP: red >77 and 1.2 x green <red <1.5 x green; strong ALP: red >77 and red >1.5 x green) [44].

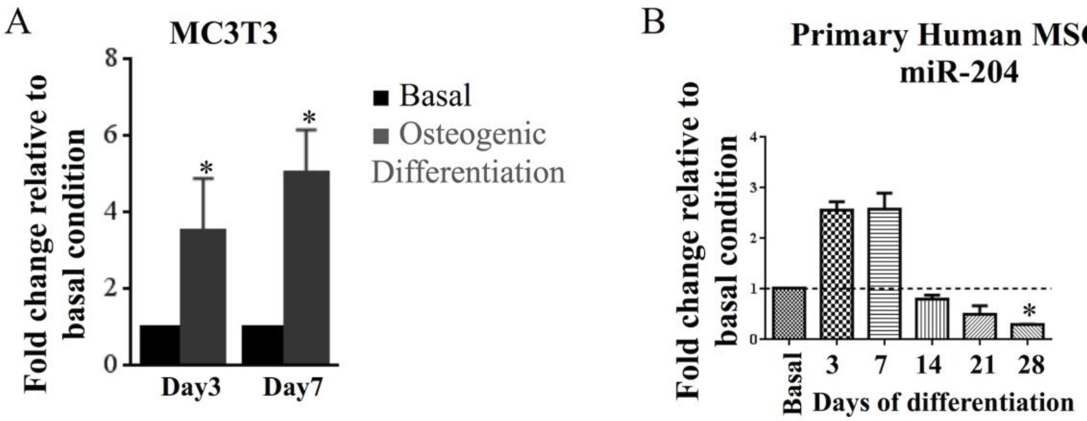

Supplementary Figure 1. mir-204 expression levels. (A) mmu- miR-204 expression levels of MC3T3 cells grown under osteogenic differentiation stimuli (osteogenic differentiation) were compared with miRNA expression levels of cells grown without osteogenic differentiation stimuli (basal), after 3 and 7 days in culture. Values are representative of 3 independent experiments (mean±SD; *P<0,05, Student t test). (B) hsa-miR-204 expression level of 2 primary human MSC grown under osteogenic differentiation stimuli (osteogenic differentiation) was compared with miRNA expression levels of cells grown without osteogenic differentiation stimuli (basal) after 3, 7, 14, 21 and 28 days in culture (mean±SEM; *P<0,05, Student t test). U6 was used as reference gene.

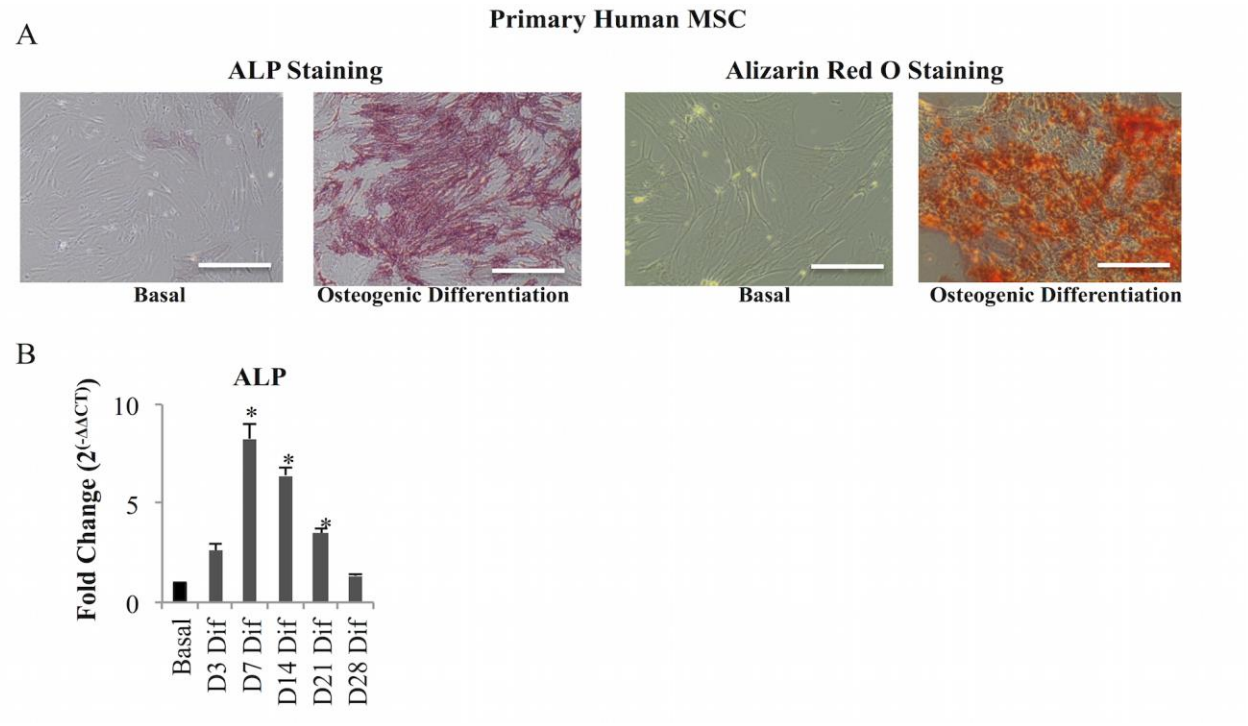

Supplementary Figure 2. Osteogenic differentiation in human primary MSC. Osteogenesis was induced with dexamethasone, β-glycerophosphate and ascorbic acid. (A) ALP staining detected ALP activity in cells grown with osteogenic differentiation but not with basal media (without osteogenic differentiation supplements); Alizarin Red O staining detected presence of calcium deposits (mineralization) in cells grown in osteogenic differentiation media but not in basal media (10X, scale: 100 µm). (B) ALP mRNA level was measured by quantitative real-time PCR in duplicates. GAPDH was used as reference control. Expression levels at day 3 (D3), day 7 (D7), day 14 (D14), day 21 (D21) and day 28 (D28) of differentiation (Dif) were normalized to expression levels of cells grown in basal conditions for the same time points (mean±SD; *P<0,05, Student t test).

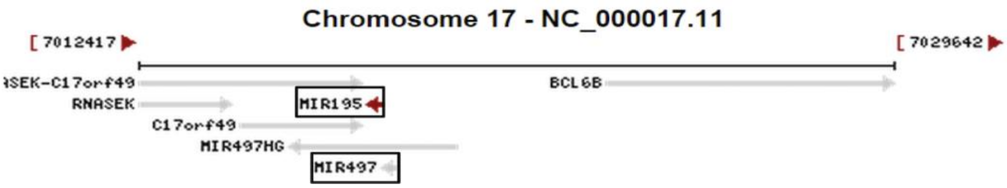

Supplementary Figure 3. Location and position of miR-195 and miR-497 in the human chromosome #17.

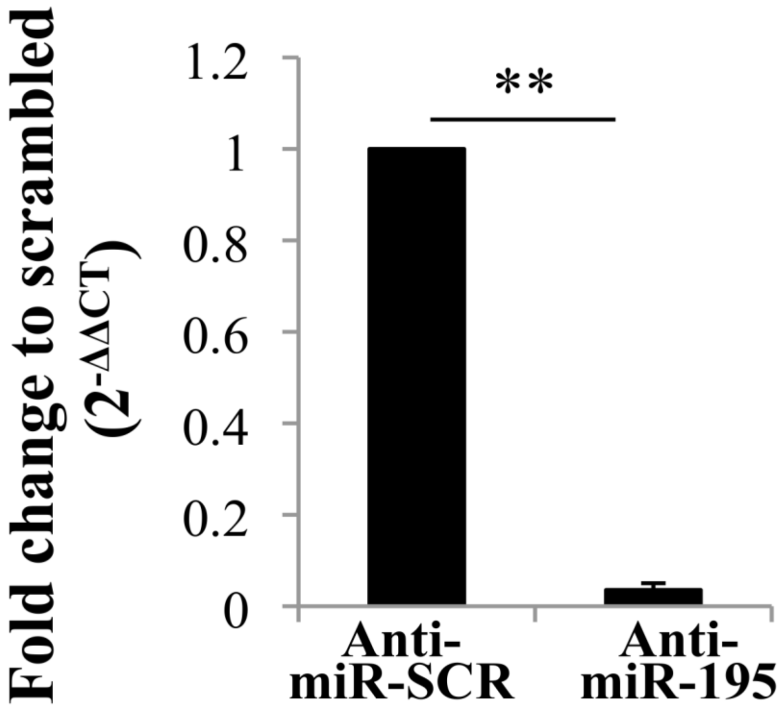

Supplementary Figure 4. Evaluation of miR-195 expression levels by quantitative real-time PCR 48h after MSC transfection with anti-miR-195 or SCR negative control (*P<0.05, Student t test).

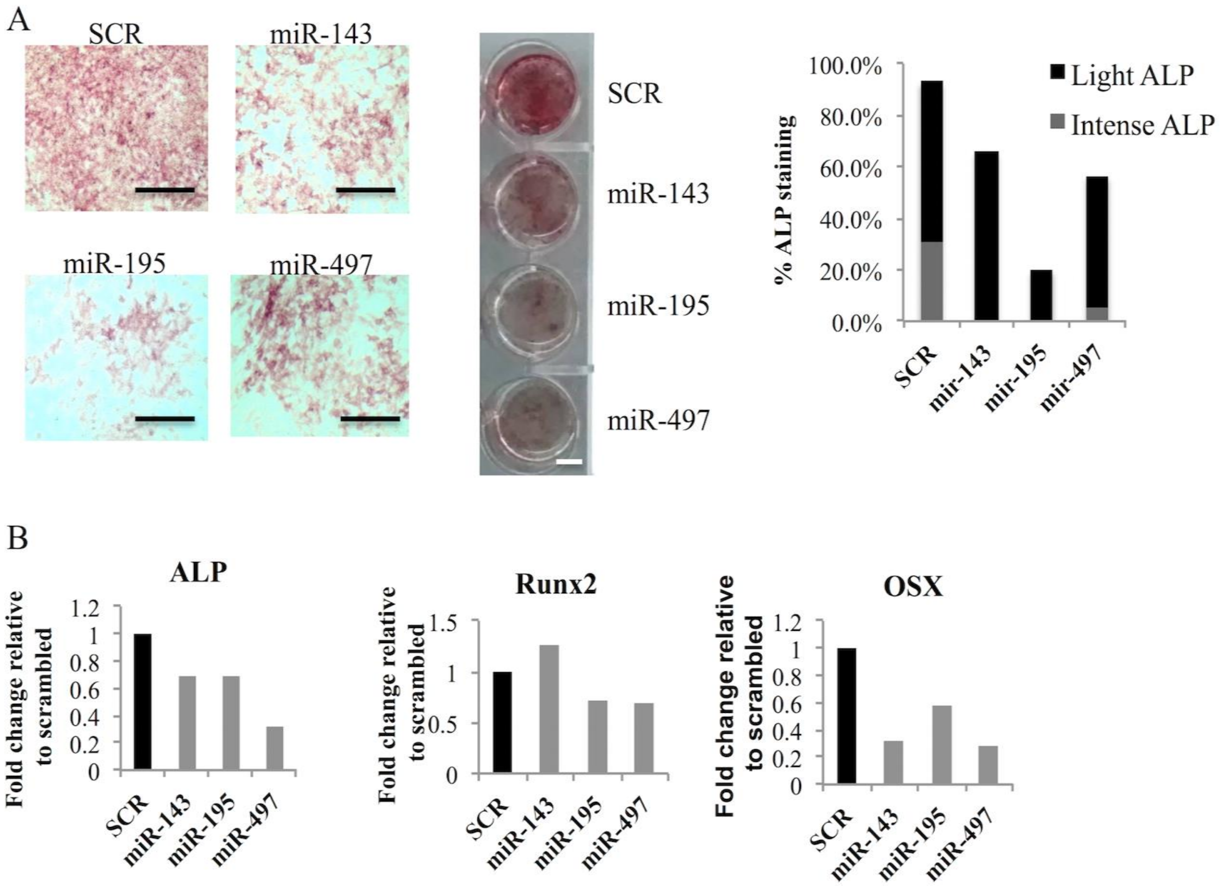

Supplementary Figure 5. miR-195, miR-497 and miR-143 decrease osteogenesis in MC3T3 cells. (A) ALP staining 7 days after MC3T3 cells transfection with scrambled negative control (SCR), miR-195, miR-497 or miR-143. Microscope and photographic images are shown and ALP levels were quantified (5X, microscope scale: 50 µm; photography scale: 2 mm). (B) ALP, RUNX2 and OSX expression levels in MC3T3 cells transfected with either SCR, miR-195, miR-497.

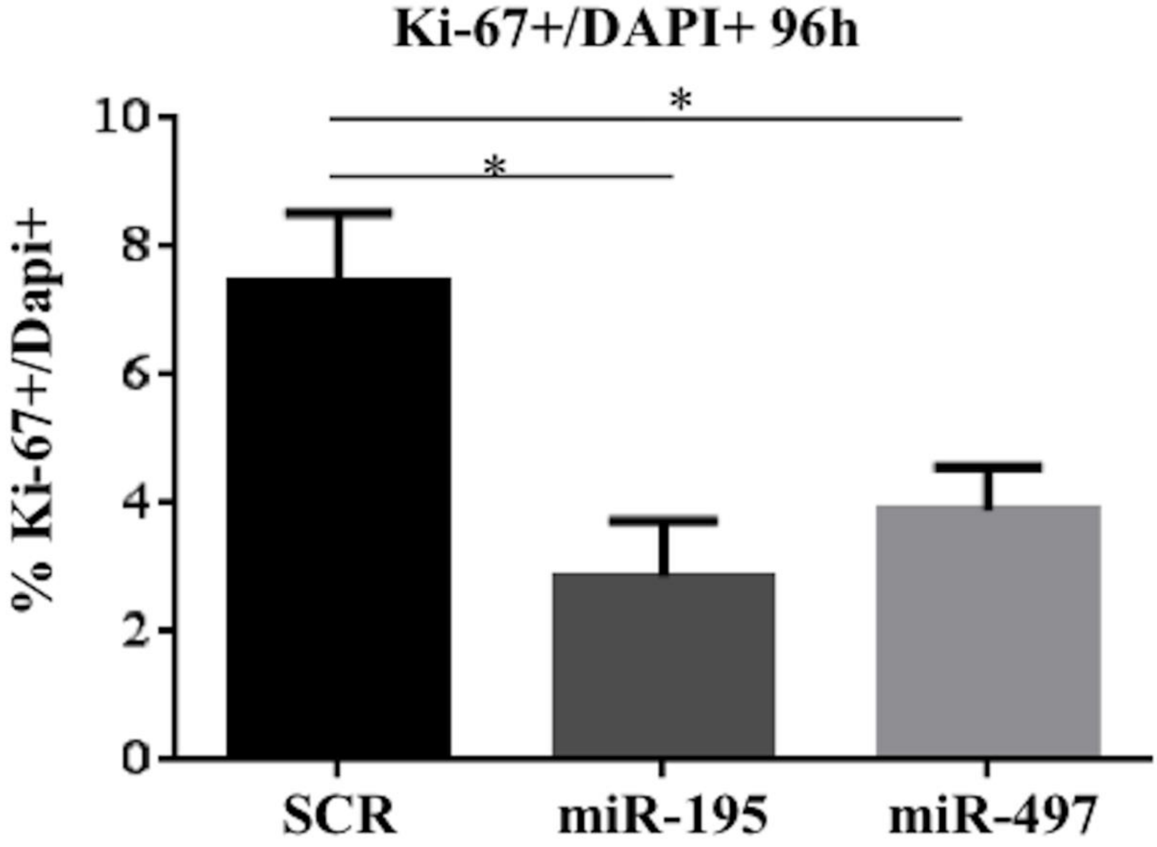

Supplementary Figure 6. Evaluation of the proliferation marker Ki-67 in miR-195 and miR-497 electroporated human primary MSC. Percentage of cells in proliferation (Ki-67+/DAPI+) was decreased in MSC over-expressing miR-195 and mir-497 compared with scrambled after 96 hours (*P<0,05, one-way ANOVA). Graphic represents 2 independent experiments with at least 5 different images per condition with a minimum of 100 DAPI+ nuclei each.

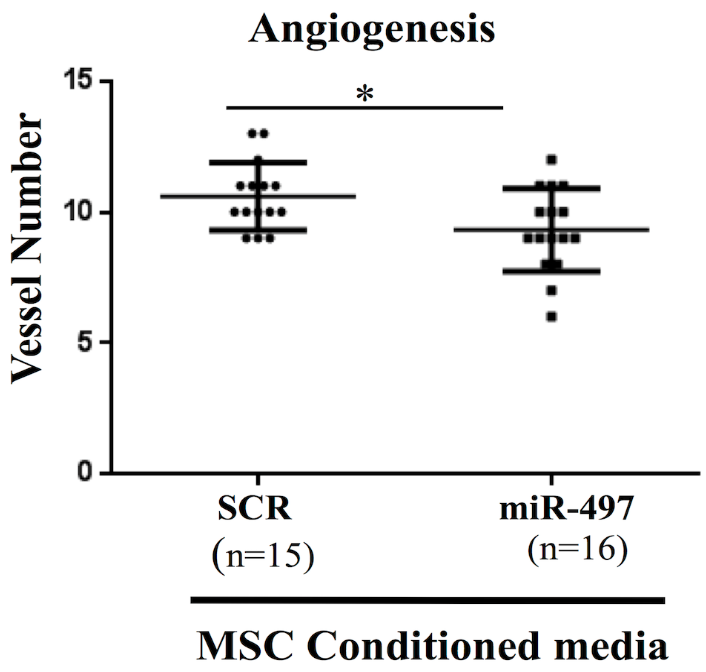

Supplementary Figure 7. In vivo Chick Chorioallantoic Membrane (CAM) Assay. Number of vessels in the CAM after 72 hours incubation with SCR-electroporated MSC condition media or miR-497-electroporated MSC condition media (mean±SD, *P<0.05, Student t test). Graphic includes results from 2 independent replicates, in a total of 31 analyzed eggs.

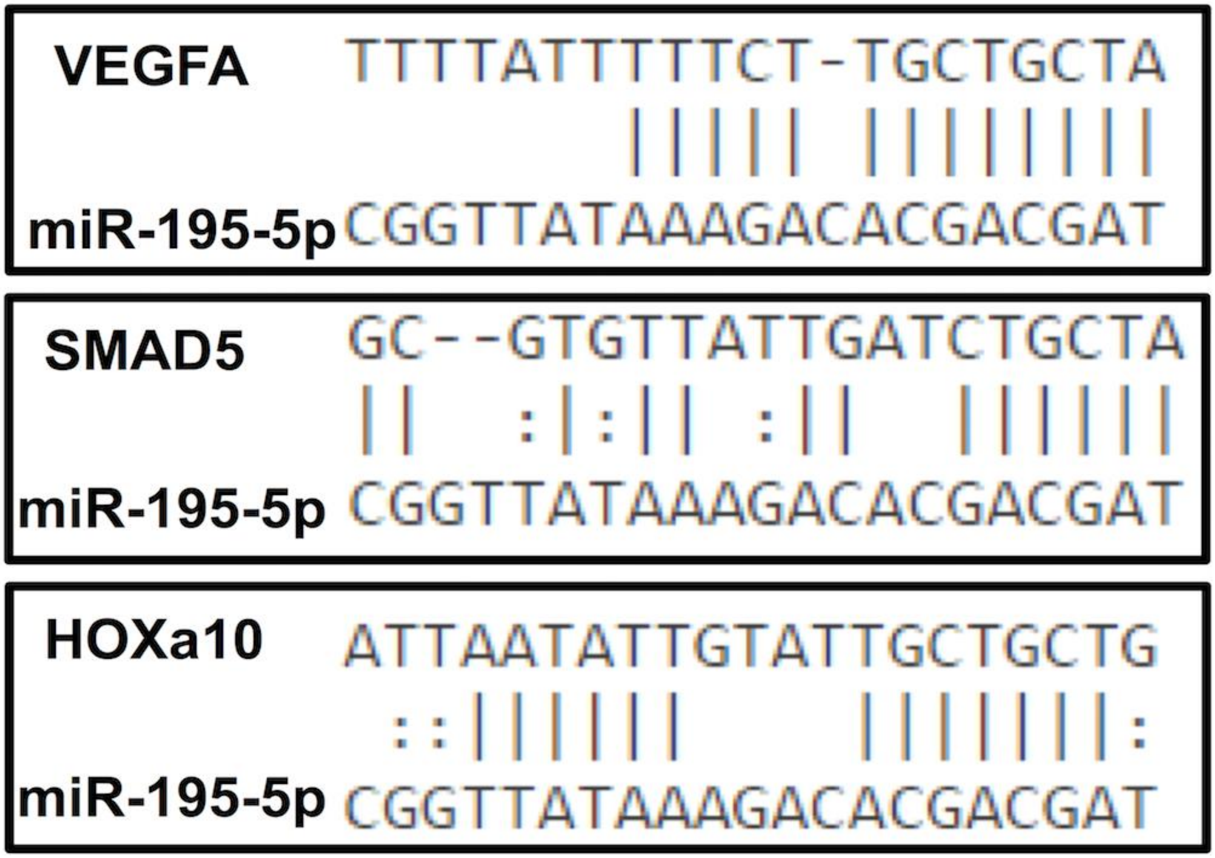

Supplementary Figure 8. *In silico* predictions of miR-195 binding site in VEGF, SMAD5 and HOXA10 mRNA (RNA22 v2 microRNA target detection, https://cm.jefferson.edu/rna22v2/).

Supplementary Table 1- Microarray data with the microRNAs differently expressed in MC3T3 cells grown with osteogenic supplements compared to control (microRNAs where the absolute value of the log fold change is larger than 0.4 were considered). MicroRNAs selected to be tested by RT-qPCR are shown in red colour.

Day3

Annotation	AvgHy3	Basal Control	Osteogenic Treatment	logFC
mmu-miR-3473b	8,662	-0,096	-1,133	-1,04
mmu-miR-5109	10,322	-0,091	-0,827	-0,74
mmu-miR-3100-3p	7,115	0,035	-0,517	-0,55
mmu-miR-218-5p	7,528	0,437	-0,081	-0,52
mmu-miR-2137	8,638	-0,680	-1,145	-0,46
mmu-miR-378b	7,639	-0,082	-0,530	-0,45
mmu-miR-378a-3p	7,870	0,049	-0,379	-0,43
mmu-miR-711	6,495	-0,455	-0,864	-0,41
mmu-miR-335-5p	9,667	-0,444	0,029	0,47
mmu-miR-450a-5p	6,271	0,205	0,684	0,48
mmu-miR-29a-5p	7,485	-0,023	0,486	0,51
mmu-miR-542-3p	7,352	-0,283	0,324	0,61
mmu-miR-455-3p	9,340	-0,623	-0,017	0,61
mmu-miR-29c-3p	7,351	-0,674	-0,033	0,64
mmu-miR-29b-3p	9,382	-0,268	0,374	0,64
mmu-miR-21a-5p	11,190	-0,283	0,457	0,74
mmu-miR-29a-3p	11,324	-0,700	0,225	0,92
mmu-miR-204-5p	8,909	-0,713	0,417	1,13

Day7

Annotation	AvgHy3	Basal Control	Osteogenic Treatment	logFC
mmu-miR-2137	8,638	0,880	-0,400	-1,28
mmu-miR-762	7,527	0,768	-0,454	-1,22
mmu-miR-711	6,495	0,712	-0,460	-1,17
mmu-miR-25-5p	6,941	0,605	-0,465	-1,07
mmu-miR-3473b	8,662	0,588	-0,391	-0,98
mmu-miR-5109	10,322	0,778	-0,097	-0,87
mmu-miR-218-5p	7,528	0,603	-0,137	-0,74
mmu-miR-378b	7,639	0,371	-0,299	-0,67
mmu-miR-143-3p	11,039	0,537	-0,128	-0,67

mmu-miR-195a-5p	8,922	0,679	0,051	-0,63
mmu-miR-378a-3p	7,870	0,469	-0,135	-0,60
mmu-miR-99a-5p	9,149	0,363	-0,233	-0,60
mmu-miR-3102-5p	6,884	0,484	-0,110	-0,59
mmu-miR-665-3p	6,132	0,429	-0,155	-0,58
mmu-miR-101a-3p/mmu-miR-101c	6,325	0,435	-0,137	-0,57
mmu-miR-5116	8,297	0,415	-0,142	-0,56
mmu-miR-335-5p	9,667	0,616	0,064	-0,55
mmu-miR-290-5p	8,891	0,391	-0,153	-0,54
mmu-miR-100-5p	8,954	0,257	-0,275	-0,53
mmu-miR-33-5p	7,504	0,738	0,248	-0,49
mmu-miR-125b-5p	14,374	0,287	-0,196	-0,48
mmu-miR-3090-5p	6,628	0,447	-0,031	-0,48
mmu-miR-101a-3p	8,133	0,734	0,306	-0,43
mmu-miR-1900	8,490	0,466	0,077	-0,39
mmu-miR-497-5p	6,197	0,237	-0,150	-0,39
mmu-miR-3970	7,605	-0,174	0,214	0,39
mmu-miR-489-5p	7,341	-0,517	-0,127	0,39
mmu-miR-712-5p	7,244	-0,100	0,301	0,40
mmu-miR-3096a-5p	9,386	-0,544	-0,143	0,40
mmu-miR-3096b-5p	9,328	-0,638	-0,219	0,42
mmu-miR-5117-3p	10,548	-0,179	0,261	0,44
mmu-miR-29b-1-5p	7,304	0,226	0,682	0,46
mmu-miR-17-5p	8,093	-0,234	0,233	0,47
mmu-miR-199a-5p	12,083	-0,124	0,368	0,49
mmu-miR-30b-3p	6,860	-0,186	0,308	0,49
mmu-miR-92a-3p	8,091	-0,636	-0,135	0,50
mmu-miR-5099	11,116	-0,334	0,182	0,52
mmu-miR-214-3p	11,786	-0,376	0,141	0,52
mmu-miR-3096a-3p	10,182	-0,449	0,070	0,52
mmu-miR-214-5p	8,252	-0,317	0,225	0,54
mmu-miR-20b-5p	6,481	-0,427	0,118	0,54
mmu-miR-140-5p	10,441	0,000	0,571	0,57
mmu-miR-3084-3p	10,683	-0,282	0,288	0,57
mmu-miR-29a-5p	7,485	0,038	0,614	0,58
mmu-miR-20a-5p	9,015	-0,191	0,389	0,58
mmu-miR-3069-5p	7,167	-0,421	0,182	0,60
mmu-miR-146a-5p	6,458	0,053	0,656	0,60
mmu-miR-3096a-3p/mmu-miR-3096b-3p	8,305	-0,741	-0,134	0,61

mmu-miR-3069-3p	8,168	-0,394	0,234	0,63
mmu-miR-21a-5p	11,190	-0,251	0,393	0,64
mmu-miR-1949	9,262	-0,418	0,267	0,68
mmu-miR-29c-3p	7,351	-0,296	0,404	0,70
mmu-miR-140-3p	11,507	-0,210	0,582	0,79
mmu-miR-221-3p	8,231	-0,044	0,925	0,97
mmu-miR-29b-3p	9,382	-0,398	0,595	0,99
mmu-miR-29a-3p	11,324	-0,593	0,406	1,00
mmu-miR-222-3p	8,708	-0,264	0,828	1,09
mmu-miR-146b-5p	8,228	0,026	1,215	1,19
mmu-miR-204-5p	8,909	-0,795	0,845	1,64

Supplementary Table 2- Sequences of miR-195 and miR-497 mature sequences and stem-loop sequences according to miRBase (http://www.mirbase.org/).

**Mature miRNA**	**miRBase accession number**	**Sequence**
hsa-miR-195-5p	MIMAT0000461	UAGCAGCACAGAAAUAUUGGC
hsa-miR-497-5p	MIMAT0002820	CAGCAGCACACUGUGGUUUGU
**miRNA Stem-loop**	**miRBase accession number**	**Sequence**
hsa-mir-195	MI0000489	AGCUUCCCUGGCUCUAGCAGCACAGAAAUAUUGGCACAGGGAAGCGAGUCUGCCAAUAUUGGCUGUGCUGCUCCAGGCAGGGUGGUG
hsa-mir-497	MI0003138	CCACCCCGGUCCUGCUCCCGCCCCAGCAGCACACUGUGGUUUGUACGGCACUGUGGCCACGUCCAAACCACACUGUGGUGUUAGAGCGAGGGUGGGGGAGGCACCGCCGAGG

Supplementary Table 3- Primer sequences used for quantitative Real-Time PCR and primers used to generate PGL3 constructs for luciferase assays and to generate deletions in the miRNA-binding site.

	Sequences
qRT-PCR	
OPN_Fw_Human	TCACCAGTCTGATGAGTCTCAC
OPN_Rv_Human	CAGGTCTGCGAAACTTCTTAGAT
OPN_Fw_Mouse	GATGAACAGTATCCTGATGCC
OPN_Rv_Mouse	TTGGAATGCTCAAGTCTGTG
ALP_Fw_Human	GACGGACCCGTCACTCTC
ALP_Rv_Human	GTGCCCGTGGTCAATTCT
ALP_Fw_Mouse	CGGACATCATGAGGGTAAGG
ALP_Rv_Mouse	GAGACATTTTCCCGTTCACC
RUNX2_Fw_Human and mouse	CCTGAACTCTGCACCAAGTC
RUNX2_Rv_Human and mouse	GAGGTGGCAGTGTCATCATC
OSX_Fw_Mouse	CCTCTGCGGGACTCAACAAC
OSX_Rv_Mouse	TGCCTGGACCTGGTGAGATG
GAPDH_Fw_Human	CGTCTTCACCACCATGGAGA
GAPDH_Rv_Human	CGGCCATCACGCCACAGTTT
GAPDH _Fw_Mouse	GACTTCAACAGCAACTCCCAC
GAPDH_Rv_Mouse	TCCACCACCCTGTTGCTGTA
VEGFA_Fw_Human	CTACCTCCACCATGCCAAGT
VEGFA_Rv_Human	GCAGTAGCTGCGCTGATAGA
SMAD5_Fw_Human	CCAGCAGCTGCAGCCTCAAAAT
SMAD5_Rv_Human	TGCCGGTGATATTCTGCTCCCCAA
HOXA10_Fw_Human	CTTCCGAGAGCAGCAAAGC
HOXA10_Rv_Human	TCTGGTGCTTCGTGTAGGG
Primers for PGL3 construct	
VEGFA_XbaI_Fw	CGTCTAGACACCATCACCATCGACAGAA
VEGFA_XbaI_Rv	CGTCTAGATGTATGTGGGTGGGTGTGTC
SMAD5_XbaI_Fw	CGTCTAGATTCAGTGTAATGTGACTTCATGCT
SMAD5_XbaI_Rv	CGTCTAGATGTCACCCATAAAAGTGCAAA
HOXA10_XbaI_Fw	CGTCTAGAAGAAGGGAGACATTGTTTGGA
HOXA10_XbaI_Rv	CGTCTAGAAAAGCTTCATTCCACAGCTTTT
Primers for mutagenesis	
VEGF_Mutagenesis_Fw	GGAATTGGATTCGCCATTTTATTTTGCTAAATCACCGAGC
VEGF_Mutagenesis_Rv	GCTCGGTGATTTAGCAAAATAAAATGGCGAATCCAATTCC

SMAD5_Mutagenesis_Fw	ACAGCCTTGTTCAACTATGTTTTTGGACAATGTTGCAAGAAC
SMAD5_Mutagenesis_Rv	GTTCTTGCAACATTGTCCAAAAACATAGTTGAACAAGGCTGT
HOXA10_Mutagenesis_Fw	CACGCACAGCAGCAATACAATTTATTCTGATTTAAGATTAGAAGTAAAT
HOXA10_Mutagenesis_Rv	ATTTACTTCTAATCTTAAATCAGAATAAATTGTATTGCTGCTGTGCGTG

Restriction sites for endonucleases are underlined.
